# Plantar fasciitis

**DOI:** 10.1308/003588412X13171221592456

**Published:** 2012-05

**Authors:** S Cutts, N Obi, C Pasapula, W Chan

**Affiliations:** ^1^James Paget University Hospitals NHS Foundation Trust,UK; ^2^Cambridge University Hospitals NHS Foundation Trust,UK; ^3^Queen Elizabeth Hospital King’s Lynn NHS Foundation Trust,UK; ^4^Peterborough and Stamford Hospitals NHS Foundation Trust,UK

**Keywords:** Plantar fasciitis, Heel pain, Extracorporeal shockwave therapy, Surgical release, Steroid injections

## Abstract

**INTRODUCTION:**

In this article we look at the aetiology of plantar fasciitis, the other common differentials for heel pain and the evidence available to support each of the major management options. We also review the literature and discuss the condition.

**METHODS:**

A literature search was performed using PubMed and MEDLINE®. The following keywords were used, singly or in combination: ‘plantar fasciitis’, ‘plantar heel pain’, ‘heel spur’. To maximise the search, backward chaining of reference lists from retrieved papers was also undertaken.

**FINDINGS:**

Plantar fasciitis is a common and often disabling condition. Because the natural history of plantar fasciitis is not understood, it is difficult to distinguish between those patients who recover spontaneously and those who respond to formal treatment. Surgical release of the plantar fascia is effective in the small proportion of patients who do not respond to conservative measures. New techniques such as endoscopic plantar release and extracorporeal shockwave therapy may have a role but the limited availability of equipment and skills means that most patients will continue to be treated by more traditional techniques.

Pain at the medial plantar prominence of the calcaneum impacts on the lives of millions of people around the world. It is estimated that 7% of people over 65 years of age report tenderness in the heel, and that the diagnosis and treatment of plantar heel pain accounts for over 1 million visits to physicians per year in the US.^1,2^

Patients usually complain of pain at the anteromedial prominence of the calcaneum. The pain is exacerbated by passive dorsiflexion of the toes. Symptoms may have been present for weeks or months at the time of presentation. The pain is worse when first standing after rest, typically early in the morning. Once the patient starts walking, the pain tends to recede. The pain eases but never fully resolves throughout the course of the day and is exacerbated by activities such as prolonged walking or exercise, particularly on hard surfaces.^3^ By the time of presentation, the patient may have already tried over the counter shoe and heel inserts.

Obesity and reduced ankle dorsiflexion are recognised risk factors for the condition.^3^ Plantar fasciitis has also been shown to be associated with biomechanical abnormalities in the foot such as a tight Achilles tendon, pes cavus and pes planus.^4^ Patients with some seronegative spondylarthropathies and gout may have an increased incidence of plantar fasciitis. While most cases of plantar fasciitis respond to conservative management and the passage of time, around 1% of patients will require surgery.

## Anatomy and function of the plantar fascia

The plantar fascia is a broad band of connective tissue that supports the arch of the foot. It includes a thick central component and thinner medial and lateral components. Functionally, the plantar fascia provides a windlass effect on the sole of the foot and helps maintain the longitudinal arch. It attaches proximally to the medial tubercle of the calcaneus. Extending distally, it divides into five digital bands that insert to the base of the periosteum of the proximal phalanx of each toe and the metatarsal heads. Fibres from the plantar fascia also blend in with the dermis, the transverse metatarsal ligament and the flexor sheath.

Hicks showed that the plantar fascia is tensioned during the latter weight bearing stage and, as the metatarsophalangeal joints dorsiflex, this applies a tractional force at its point of insertion on the calcaneum.^5^ He named this the windlass effect. It also plays a dynamic role during the gait cycle where it elongates during the stance phase, storing potential energy during the process. It locks the midfoot during toe-off to provide a rigid structure for propulsion. The plantar fascia then passively contracts, converting the previously stored potential energy into kinetic energy and aiding acceleration.

## Mechanism of injury

Remarkably for such a common condition, the underlying pathological changes in plantar fasciitis are not understood. Even the term plantar fasciitis is something of a misnomer since the plantar fascia is an aponeurotic rather than a fascial layer.

It has been suggested that plantar fasciitis represents a form of tennis elbow at the heel with the condition being caused by repetitive microtrauma at the point of insertion.^6^ Inflammation triggered by microtrauma might also explain why the condition sometimes responds to local steroid injection.

At night, the foot usually falls into a plantar flexed position and when a patient alights from bed in the morning, the foot moves into dorsiflexion during walking. The plantar fascia slightly contracts in bed and the initial stretching associated with early morning waking is probably responsible for initiation pain.

In older patients, histology on the plantar fat pad shows gradual atrophy. These changes may be accelerated by repeated steroid injections. Lemont *et al* looked at 50 cases of plantar fasciitis that had been treated with heel spur surgery and failed to find histological evidence of inflammation.^7^ Histologically, the condition is a fasciosis.

In view of such findings, it is perhaps surprising that so many patients seem to respond to local steroid injections and oral anti-inflammatory drugs. However, this paradox is hardly unique to plantar fasciitis. Both lateral and medial epicondylitis respond to similar treatments in spite of the inability of modern histologists to find inflammatory changes at the site of tenderness. It must also be remembered that histology is not obtained on all patients with heel pain and the absence of inflammation in the subgroup (less than 1%) that comes to surgery may not be an accurate representation of plantar fasciitis patients as a whole.

A branch of the lateral plantar nerve passes between the heel spur and the deep surface of the flexor digitorum brevis. This structure has also been implicated in plantar pain of this kind. This pain can be more focal and does not get worse with passive dorsiflexion of the toes. The pain may also be more diffuse. In a series of patients presenting with heel pain, a neurogenic cause for plantar pain was identified in a significant proportion.^8^ Two of their patients were also shown to have S1 nerve root entrapment, suggesting a possible double crush syndrome. It has been recommended that in those cases where the patient is suffering from lateral tenderness, the deep fascial edge of the abductor hallucis should be released during surgery.

## Differential diagnoses

While 80% of patients with heel pain are suffering from plantar fasciitis, there are a number of other differential diagnoses. Ankylosing spondylitis, Reiter’s syndrome and osteoarthritis can all produce these symptoms. When symptoms are bilateral, rheumatoid arthritis becomes more likely in women. In men, however, one should consider ankylosing spondylitis or Reiter’s syndrome. An abscess in the soft tissues is more likely in patients with diabetes mellitus. Constitutional symptoms such as weight loss, night pain and fever are suggestive of neoplasia or infections, particularly in neuropathic patients, although primary neoplasia in the foot is extremely rare. Other differential diagnoses include entrapment of the first branch of the lateral plantar nerve or the medial calcaneal nerve,^9^ S1 radiculopathy and occult fracture.

## Investigations

First and foremost, the diagnosis of plantar fasciitis is a clinical one. Further investigations should be tailored depending on the clinical picture. Plain x-ray is the most commonly requested investigation. X-ray shows a calcaneal spur in 50% of patients. The spur is not related to the plantar fascia and is the attachment of the quadratus plantae muscle. Thirteen per cent of heels x-rayed for conditions other than plantar fasciitis also have a plantar spur and many authors regard the heel spur as an incidental finding.^10^

Technetium bone scintigraphy is positive in plantar fasciitis, with the maximum area of uptake at the point of maximum tenderness on the heel. Bone scintigraphy also shows an area of increased uptake in the presence of an occult fracture. Electromyography may be helpful if a neurogenic cause is suspected such as S1 nerve root entrapment, tarsal tunnel syndrome or entrapment of the lateral plantar nerve. Magnetic resonance imaging is not a routine investigation in plantar fasciitis but can identify other soft tissue lesions such as soft tissue tumours or the marrow oedema associated with infection or if an occult fracture is suspected.^11^ Depending on the overall clinical picture, the physician may also perform blood tests such as a white cell count, human leucocyte antigen B27, antinuclear antibodies and uric acid, particularly in the younger patients or with those patients who have bilateral heel pain.

## Treatment of plantar fasciitis

### Lifestyle modification

The vast majority of patients recover without surgery although the indolent course of this condition is often frustrating. Patient education into the expectation and duration of treatment from the outset is important. Plantar fasciitis would appear to be an overuse phenomenon and, in the first instance, avoiding high impact activities seems sensible.

### Oral analgesia and non-steroidal anti-inflammatory drugs

Oral analgesics are also traditional. In a randomised, prospective, placebo controlled trial, 29 plantar fasciitis sufferers were randomised to treatment with heel cord stretching, viscoelastic heel cups and night splinting to a placebo or a non-steroidal anti-inflammatory drugs (NSAID) group.^12^ It was found that the NSAID group had improved pain relief and disability, especially in the interval between two and six months. However, the advantages of NSAIDs over placebo were not statistically significant, possibly because of the small scale of the trial. In patients with seronegative disease, NSAIDS can produce a dramatic response.

### Steroid injections

Steroid injections are often effective in the short term^13^ although they have been shown to cause fat pad atrophy and, very occasionally, they may precipitate rupture of the plantar fascia.^14^ One case series of six athletes with rupture of the plantar fascia noted five had previously received steroid injection.^15^ The risk of rupture is reduced if the injection is given on the medial side of the heel, superior to the plantar fascia.

### Stretching

Stretching is an easy implementable treatment option. A randomised controlled trial has investigated the role of specific Achilles tendon versus plantar fascia stretching exercises in patients with established chronic plantar fasciitis.^16^ This involved an eight-week programme supplemented by celecoxib for the first three weeks. The short-term outcome demonstrated superior results relating to pain using the Foot Function Index in the plantar fascia specific stretching group compared with the Achilles stretching group. The two-year follow-up via questionnaire demonstrated 94% of respondents reported decreased pain and 92% reported total satisfaction or satisfaction with minor reservations although no significant difference separated the two groups at this late stage.^17^

### Orthotic devices

Night splints hold the foot in a neutral position, preventing the contracture of the fascia during sleep, which helps to alleviate symptoms in the morning according to observational studies. However, there has been no randomised controlled trial to prove that symptoms are alleviated. Casting is a prolonged version of this and forces complete rest.

Heel inserts are quite popular and can be beneficial in relieving heel pain. Wolgin *et al* showed that after six months, 82% of patients had responded to time and conservative therapy.^18^ A plastic heel insert has also been used to good effect.^19^

The response to conservative measures is usually very good. A case series of 116 patients in which only two patients required surgery suggested that the proportion of patients requiring surgery is of the order of 1–2%.^20^

### Botulinum toxin

Work looking into the effect of botulinum toxin injections to treat plantar fasciitis has shown apparently good effect although this is still far from mainstream.^21^

### Extracorporeal shockwave therapy

In recent years, some units have used extracorporeal shockwave therapy (ESWT). Although the technique has been investigated extensively, it is mostly performed in specialist centres. Proponents of ESWT believe that the shockwaves cause microdisruption of the thickened plantar fascia, resulting in an inflammatory response, revascularisation and recruitment of growth factors and therefore a soft tissue reparative response.^22^ ESWT should, perhaps, be regarded as an end stage treatment for those patients who have failed conservative measures and are reluctant to have open surgery.^23,24^ A double-blind randomised controlled trial showed radial ESWT to be better than placebo in recalcitrant patients.^25^ When successful and when compared with operative intervention, ESWT can be seen to avoid the risks associated with surgery.

### Protein rich plasma

At present there is a lack of good quality evidence to support the efficacy of protein rich plasma injections in the treatment of plantar fasciitis. The patient’s own plasma is spun in a centrifuge and a specific fraction is then reinjected into the tender area. The treatment is discussed widely on the internet although there are still no clinical trials on PubMed to report.

### Surgery

Patients not responding to conservative measures after a year or more may require surgery. There is controversy as to which procedures are most likely to produce a good result, in part because of the limited scale of many published series.

Less than 50% of the plantar fascia is sectioned. Any more than this risks secondary collapse of the longitudinal arch and lateral-sided foot pain.^26^ Cadaveric studies have reproduced the lateral column overload that can follow from this.^27^

Carefully selected patients probably benefit from simultaneous release of the lateral plantar nerve branch. However, there must be clear evidence of neurological symptoms to justify this.^28^

Removal of the spur is not warranted. A large cadaveric study found that in about half of cases, the bony spur is not actually in the same layer as the plantar fascia, suggesting that it does not have a causal role in the condition.^29^

Endoscopic surgery requires specialist equipment and skills, and is still not widely used.^30^ A retrospective review of 22 consecutive patients showed good or excellent results in 68% of patients.^31^ Accidental destruction of the lateral plantar nerve has been described using the endoscopic technique.

## Conclusions

Plantar fasciitis is a common and often disabling condition that benefits from appropriate treatment. Because the natural history is not fully understood, it is difficult to distinguish between those patients who have recovered spontaneously and those who have responded to formal treatment.

Surgical release of the plantar fascia is effective in the small proportion of patients who do not respond to conservative measures. Removal of the plantar heel spur remains a tradition in some units but is probably irrelevant.

New techniques such as endoscopic plantar release and ESWT may have a role but the limited availability of equipment and skills means that most patients will continue to be treated by more traditional techniques.

Treatments that appear to be effective are in part traditional since the large scale randomised trials have still not been performed. The patients labelled as suffering from heel pain are likely to represent a diverse group and those referred for surgery may represent a different cohort of people from those responding rapidly to conservative measures.
